# Simplifying the Preparation of Pollen Grains for MALDI-TOF MS Classification

**DOI:** 10.3390/ijms18030543

**Published:** 2017-03-03

**Authors:** Franziska Lauer, Stephan Seifert, Janina Kneipp, Steffen M. Weidner

**Affiliations:** 1 Department of Chemistry, Humboldt-University to Berlin, Brook-Taylor-Str. 2, 12489 Berlin, Germany; franziska.lauer@bam.de (F.L.); stephan@familie-seifert.org (S.S.); janina.kneipp@chemie.hu-berlin.de (J.K.); 2 Federal Institute for Materials Research and Testing (BAM), Richard-Willstätter-Str. 11, 12489 Berlin, Germany

**Keywords:** MALDI-TOF MS, conductive carbon tape, pollen, sample pretreatment, principal component analysis

## Abstract

Matrix-assisted laser desorption ionization time of flight mass spectrometry (MALDI-TOF MS) is a well-implemented analytical technique for the investigation of complex biological samples. In MS, the sample preparation strategy is decisive for the success of the measurements. Here, sample preparation processes and target materials for the investigation of different pollen grains are compared. A reduced and optimized sample preparation process prior to MALDI-TOF measurement is presented using conductive carbon tape as target. The application of conductive tape yields in enhanced absolute signal intensities and mass spectral pattern information, which leads to a clear separation in subsequent pattern analysis. The results will be used to improve the taxonomic differentiation and identification, and might be useful for the development of a simple routine method to identify pollen based on mass spectrometry.

## 1. Introduction

Matrix-assisted laser desorption ionization mass spectrometry (MALDI MS) has become a suitable technique to explore complex biological materials, such as small organisms (e.g., ticks, flies, mites etc.) [1,2,3], microorganisms [4,5,6], or biological particles (e.g., pollen grains) [7]. For this purpose, various sample preparation procedures have been modified, which involved grinding of organisms [8], suspension/extraction methods [3,6] or the dissection of organs [2].

Besides the digestion/extraction step, the fixation of such samples represents a crucial step for the success of the experiment. In contrast to MALDI MS of samples that are deposited on the target plate by being embedded in a matrix, or by covering tissue sections obtained from microtomes with a matrix [9,10,11], these samples have to be fixed by alternative methods. Widely known is the application of surface-enhanced laser desorption ionization time-of-flight mass spectrometry (SELDI-TOF MS) [12]. There, the target holders incorporate either modified chemical spots (hydrophobic, cationic, anionic, metal ions or hydrophilic), suited for protein expression profiling studies, or pre-activated biological surfaces designed for coupling of biomolecules [12]. A recent overview of the application of SELDI-TOF MS for the discovery of biomarkers of various forms of human cancer is given by Gopal et al*.* [13]. However, this technique is limited to the investigation of analytes with comparatively low molecular masses.

Another approach is the fixation of samples in adhesives. Kaftan et al. used epoxy glue for the fixation of flies [1]. Recently, we showed that pollen grains could be fixed in a resin typically used for atomic force microscopy (AFM) applications, without affecting the extraction of analytes or the ionization procedure in MALDI-TOF MS [14]. In this approach, we showed recently that pollen grains could be fixed without affecting the extraction of analytes or the ionization procedure in MALDI-TOF MS. This approach, however, is time-consuming, since it involves various steps. First, pollen grains are deposited on a resin film that has to be prepared on the target before. Afterwards, the target plate is exposed to solvent vapor that weakens the resin and enables the pollen grains to be fixed in the viscous film. In a subsequent step, the fixed pollen samples are exposed to formic acid atmosphere, with the purpose of extracting the analytes from the pollen grain. Finally, the layer is covered by a matrix. It becomes obvious that this approach is not applicable for fast and automated pollen monitoring. Thus, we focused our efforts on the development of alternative methods for fast pollen grain fixation for MALDI MS probing.

For pollen classification and identification, the applicability of MALDI-TOF MS was proven, combining the mass spectral data with hierarchical cluster analysis (HCA) and principal component analysis (PCA) [15]. There, almost 200 pollen samples of 74 different species from 11 genera and 2 plant orders were classified according to their taxonomic relationships, including discrimination of species that feature a very high chemical similarity [15]. The application of multivariate statistics (e.g., PCA) to mass spectral patterns yields an enormous improvement concerning taxonomic classification of the samples compared to common microscopic techniques.

In order to simplify the sample preparation process, we suggest the application of a sticky conductive carbon tape. Conductive tape was used in this context previously for the analysis of methamphetamine incorporated in hair [16]. A recent application was reported by Kajiwara et al., where spider mites were fixed on a double-sided carbon tape and analyzed by MALDI-TOF MS [8]. In this article, we demonstrate that the use of conductive carbon tape on a MALDI target facilitates the pollen sample treatment, compared to a conventional sample deposition on a steel target. Different preparation strategies are compared, e.g., regarding the influence of acid concentration in the matrix solution and additional extraction with liquid or gaseous acid. In addition, possible interferences of the formic acid used for extraction with the new target material are examined. The findings show that our approach enhances the information that can be taken from the species-specific mass peak patterns. This results in a clear differentiation in subsequent pattern analysis, which is important when analyzing pollen grains in various real mixtures.

## 2. Results

Our experiment included pollen grains from four tree species: Scots pine (*Pinus sylvestris*), Japanese bog birch (*Betula tatewakiana*), Italian aider (*Alnus cordata*) and common hazel (*Corylus avellana*). The first sample represents the plant family of *Pinaceae* in the order of *Coniferales*, the other samples belong to the plant family of *Betulaceae* in the order of *Fagales*.

**Figure 1:**
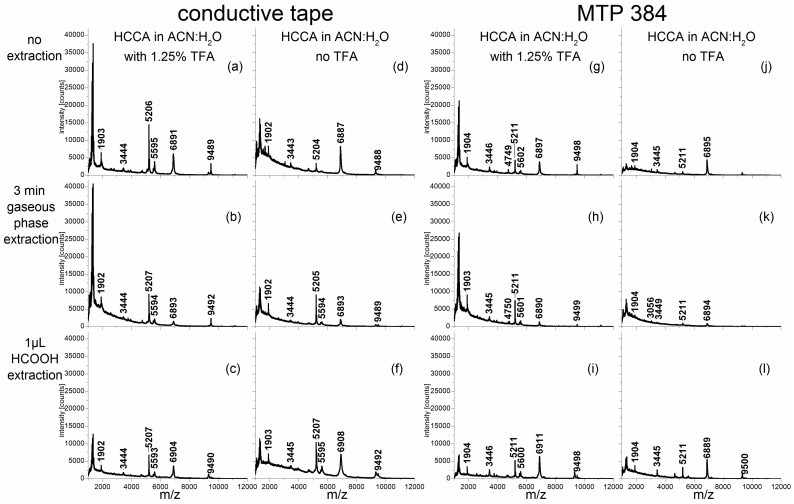
Averaged matrix-assisted laser desorption ionization time of flight (MALDI-TOF) mass spectra (*n* = 5) of *Alnus cordata* pollen grains: measured on conductive tape with trifluoroacetic acid (TFA) in the matrix solution (**a**–**c**), on conductive tape without TFA in the matrix solution (**d**–**f**), on the steel target (microtiter plate MTP 384) with TFA in the matrix solution (**g**–**i**) and on the steel target without TFA in the matrix solution (**j**–**l**) (in the rows: extraction procedure).

Spectra from all samples were recorded on conductive tape and on a stainless steel target, respectively. In Figure 1, the average spectra from five repetitive measurements obtained with different sample treatments and target materials are depicted exemplarily for *Alnus cordata*. All average spectra showed similar peak patterns, suggesting that these peak patterns are characteristic of this *Alnus cordata* sample. However, slight shifts in the accurate masses and differences in signal intensities can be detected using different sample treatments. Spectra, which were recorded on the conductive tape (Figure 1a–f) generally showed higher absolute signal intensities compared to those with the same treatment strategies measured on the steel target (Figure 1g–l). Adding trifluoroacetic acid (TFA) to the matrix solution (Figure 1a–c,g–i) also resulted in increased signal intensities, compared to the respective spectra obtained without TFA addition (Figure 1d–f,j–l). When the extraction procedure was conducted with an additional droplet deposition of formic acid onto the pollen grains (Figure 1c,f,i,l), spectra were obtained that feature low signal intensities, even though the individual peaks were clearly distinguishable. In the spectra obtained after formic acid gas phase extraction of the sample (Figure 1b,e,h,k), the background in the lower mass range as well as the absolute intensities were more intense than in the spectra gained from droplet extraction. Spectra of the pollen samples with no additional extraction step (Figure 1a,d,g,j) provided signal intensities in the lower mass range that are equal to those of the spectra obtained with gas phase extraction, whereas higher mass peaks (e.g., at *m*/*z* 6891 and *m*/*z* 9489) were more intense. The most intense spectrum showing characteristic masses of *Alnus cordata* was recorded on conductive tape using a mixture of ACN/H_2_O (1.25% TFA) as the matrix solvent and without any additional extraction steps (Figure 1a).

**Figure A1:**
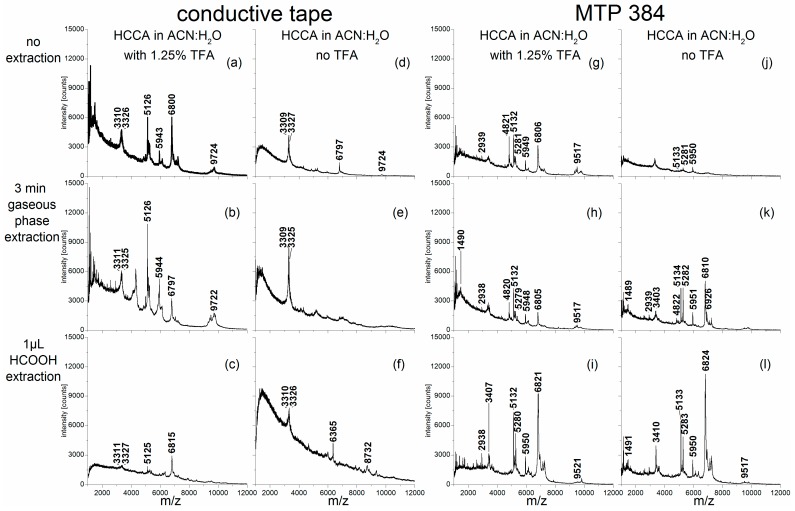
Averaged MALDI-TOF mass spectra (*n* = 5) of *Betula tatewakiana* pollen grains: measured on conductive tape with TFA in the matrix solution (**a**–**c**), on conductive tape without TFA in the matrix solution (**d**–**f**), on the steel target (MTP 384) with TFA in the matrix solution (**g**–**i**) and on the steel target without TFA in the matrix solution (**j**–**l**) (in the rows: extraction procedure).

**Figure A2:**
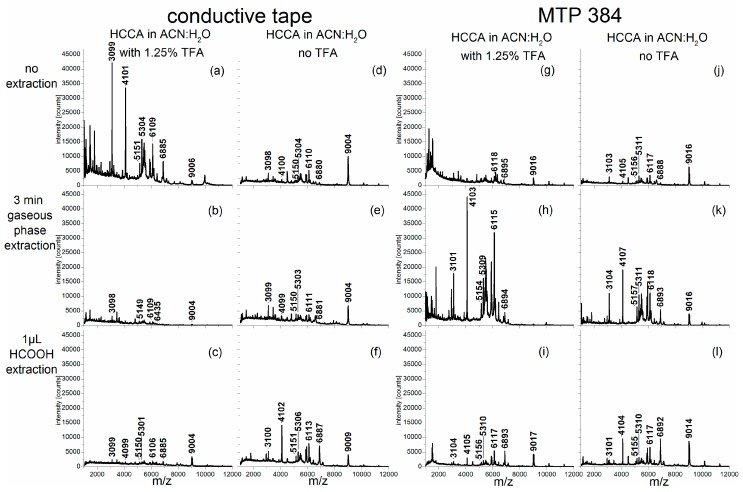
Averaged MALDI-TOF mass spectra (*n* = 5) of *Corylus avellana* pollen grains: measured on conductive tape with TFA in the matrix solution (**a**–**c**), on conductive tape without TFA in the matrix solution (**d**–**f**), on the steel target (MTP 384) with TFA in the matrix solution (**g**–**i**) and on the steel target without TFA in the matrix solution (**j**–**l**) (in the rows: extraction procedure).

**Figure A3:**
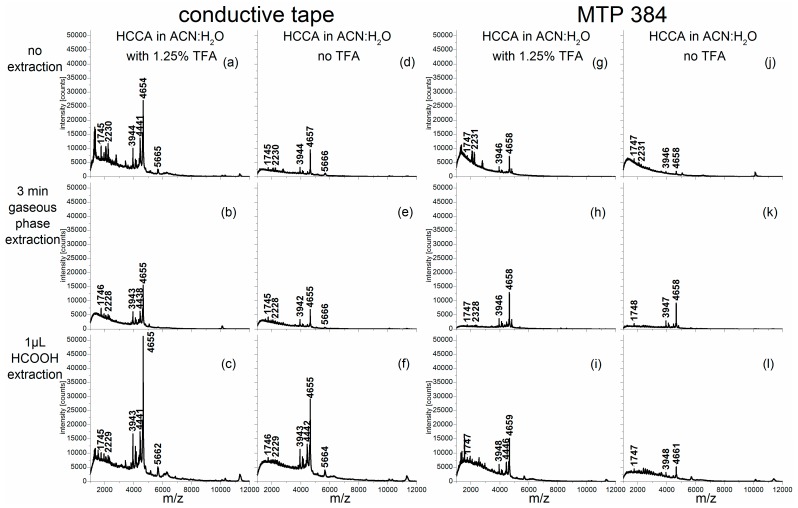
Averaged MALDI-TOF mass spectra (*n* = 5) of *Pinus sylvestris* pollen grains: measured on conductive tape with TFA in the matrix solution (**a**–**c**), on conductive tape without TFA in the matrix solution (**d**–**f**), on the steel target (MTP 384) with TFA in the matrix solution (**g**–**i**) and on the steel target without TFA in the matrix solution (**j**–**l**) (in the rows: extraction procedure).

The corresponding mass spectra of the other pollen species (*Betula tatewakiana*, *Corylus avellana* and *Pinus sylvestris*) are shown in the Appendix (Figure A1, Figure A2 and Figure A3). As indicated by the intensities of the corresponding signals, the use of a matrix solvent containing TFA promoted the extraction of analytes from the exines, i.e., the pollen grains’ outer shells, of most of these pollen samples much more than an additional extraction step using formic acid (for example compare Figure A2a,h with Figure A2c,f,i,l; or Figure A3a–c with Figure A3b,e,h,k). With the exception of the birch pollen investigations (*Betula tatewakiana*) (Figure A1), our previous findings concerning *Alnus cordata* spectra in Figure 1 were confirmed by the analysis of additional pollen species.

The study of the birch pollen sample with formic acid droplet deposition on the MTP (microtiter plate) steel target holder (Figure A1i,l) yielded intense spectra that showed a very low background in the lower mass range. In that case, the use of conductive tape in combination with a matrix solvent containing TFA and no additional extraction steps (Figure A1a) did not provide the spectra with the highest quality. Nevertheless, the spectrum also showed the characteristic peak pattern (as in Figure A1i,l) that could be analyzed by multivariate tools, as will be discussed below.

At first glance, the spectra of all four pollen species show species-specific peak patterns, which already makes a rough differentiation of species possible by comparing the spectra by eye (Figure 1 and Figure A1, Figure A2 and Figure A3). In addition to this, an objective differentiation that enables an evaluation of particular sample treatment methods for the purpose of classification and ultimately identification can be obtained by using multivariate data analysis.

Multivariate methods, such as principal component analysis (PCA) can be applied to mass spectrometric data for several reasons, e.g., for quality control, to reduce complexity of the data, and to emphasize differences within a data set that enable classification [17,18,19,20].

**Figure 2:**
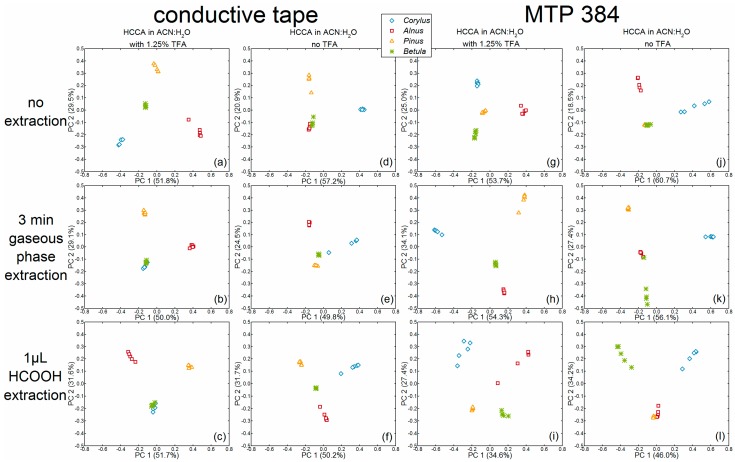
Scores and variances of the first and second principal components (PC) of the PCA (principal component analysis) of MALDI MS spectra from four pollen species (*Corylus avellana*, *Alnus cordata*, *Pinus sylvestris*, *Betula tatewakiana*) for different sample preparation techniques (rows: variation in extraction procedure ((**a**,**d**,**g**,**j**): without additional extraction); (**b**,**e**,**h**,**k**): gaseous extraction); (**c**,**f**,**i**,**l**): droplet extraction)); columns: variation in target material and matrix solution ((**a**–**c**): conductive tape with trifluoroacetic acid in the matrix solution); (**d**–**f**): CT without TFA in the matrix solution); (**g**–**i**): steel target, matrix with TFA); and (**j**–**l**): steel target, matrix without TFA)). Data pre-treatment is explained in the methods Section 4.4.

**Figure 3:**
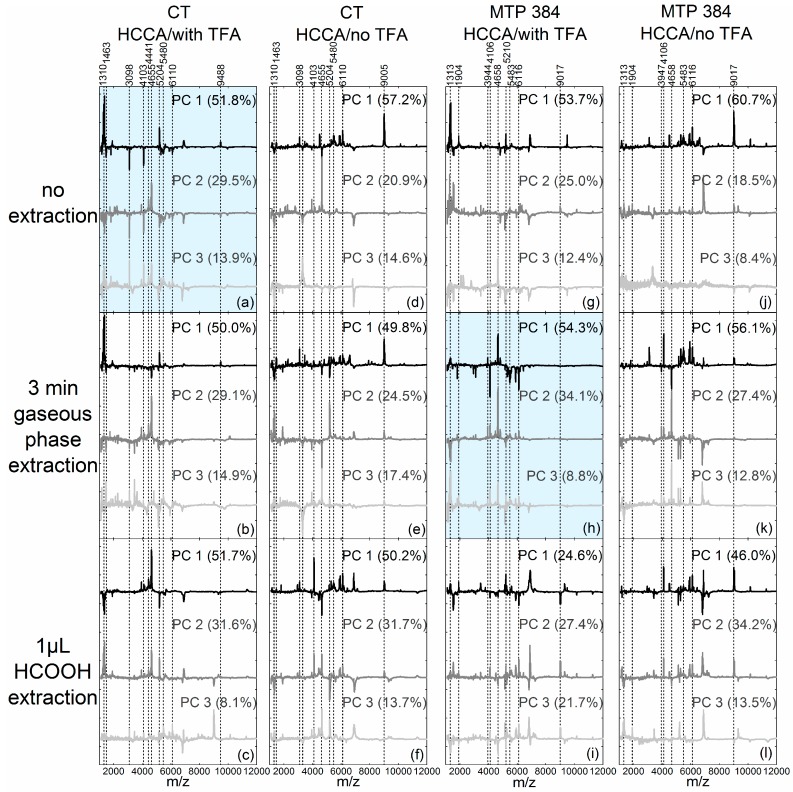
Loadings and variances (in brackets) of the first PC (top, black), the second PC (middle, gray) and the third PC (bottom, light gray) of the respective PCAs (rows: variation in extraction procedure ((**a**,**d**,**g**,**j**): without additional extraction); (**b**,**e**,**h**,**k**): gaseous extraction); (**c**,**f**,**i**,**l**): droplet extraction)); columns: variation in target material and matrix solution ((**a**–**c**): conductive tape with trifluoroacetic acid in the matrix solution); (**d**–**f**): CT without TFA in the matrix solution); (**g**–**i**): steel target, matrix with TFA); and (**j**–**l**): steel target, matrix without TFA)), for better comparison, graph (**a**,**h**) are highlighted.

We applied PCA to compare classification of pollen species upon the application of different preparation procedures and to evaluate the preparation setup by finding differences in the chemical composition of the samples. Principal components (PCs) are linear combinations of the original mass spectra and generate a new coordinate system that is based on maximum variance between the spectra [21]. They are characterized by two matrices: the loadings and the scores, as well as the percentage of original variance they are representing [22,23,24]. The scores of the first and second principal components of the PCA using the respective spectra (Figure 1 and Figure A1, Figure A2 and Figure A3) after pre-treatment (see Section 4.4 for further information) are depicted in Figure 2. In order to compare the separation of the pollen species, the scores plot of each PCA was evaluated regarding the intra- versus inter-species distances. The loadings, given in Figure 3, show the connection between the principal components and the original data and can be used for the subsequent interpretation of the scores values (Figure 2). They indicate which parts of the spectrum are relevant for the respective PC and lead to the separation of the pollen spectra in the scores plots of Figure 2.

**Figure A4:**
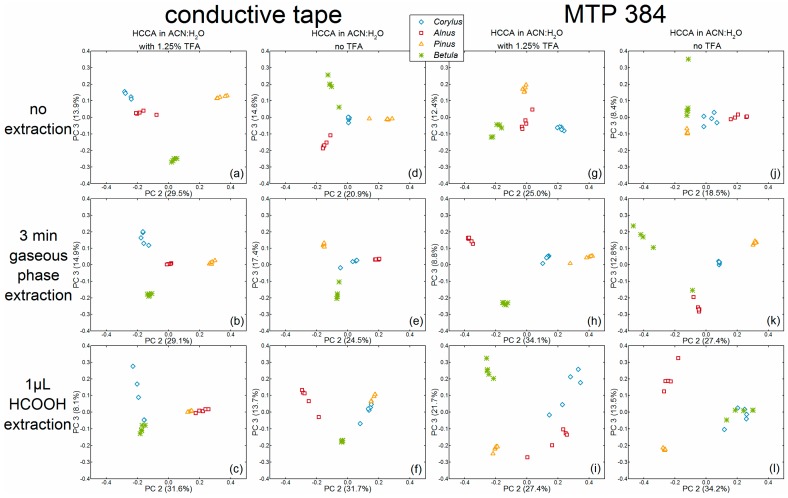
Scores and variances of the second and third principal component (PC) of the PCA of MALDI MS spectra from four pollen species (*Corylus avellana*, *Alnus cordata*, *Pinus sylvestris*, *Betula tatewakiana*) for different sample preparation techniques (rows: variation in extraction procedure ((**a**,**d**,**g**,**j**): without additional extraction); (**b**,**e**,**h**,**k**): gaseous extraction); (**c**,**f**,**i**,**l**): droplet extraction); columns: variation in target material and matrix solution ((**a**–**c**): conductive tape with trifluoroacetic acid in the matrix solution); (**d**–**f**): CT without TFA in the matrix solution); (**g**–**i**): steel target, matrix with TFA); and (**j**–**l**): steel target, matrix without TFA)). Data pre-treatment is explained in the methods Section 4.4.

The first parameter to be discussed using the PCA scores plots is the addition of TFA in the matrix solutions: PCAs conducted on spectra obtained without TFA in the matrix solution (Figure 2d–f,j–l) shared a common trait, that their respective first PC enables a separation of *Corylus avellana* spectra (light-blue diamonds) by positive values from negative or zero values of the spectra from all other species. In accord with this, the *Corylus*-typical spectral regions, e.g., from *m*/*z* 5400 to 6117 [15], yielded positive signals in the loadings of the respective first PCs (see Figure 3, 2nd and 4th column, black loadings spectra). The other species, however, cannot be differentiated clearly from each other using the scores plots in Figure 2d,e,j–l. The scores of the second and third PCs of the respective data sets, did not allow clear and accurate differentiation between the species *Alnus cordata*, *Pinus sylvestris* and *Betula tatewakiana* (see Figure A4) either.

In the PCAs of the data sets that were obtained with the addition of TFA in the matrix solution, a separation of individual pollen species using the first two PCs was possible more frequently, as indicated in the plots illustrated in Figure 2a,g,h,i. However, in contrast to the plots in Figure 2a,h, where the individual groups were clearly separated from each other, the distances of the different groups in the plot in Figure 2g were much smaller. Moreover, considering the homogeneity of each individual group in these four plots, the plot in Figure 2i especially showed very inhomogeneous distribution of the spectra of *Alnus cordata* and *Corylus avellana*. Since high intra-species variances are especially noticeable in the bottom row of Figure 2c,f,i,l they could possibly be explained by the samples treatment with the formic acid droplet method. Different to this observation, the PCAs of data obtained by additional gaseous extraction (Figure 2b,e,h,k) and by no additional extraction step (Figure 2a,d,g,j) showed no definite tendency concerning intra-species variances.

Since the scores plots in Figure 2a,h are suitable for a complete differentiation of pollen species, the underlying PCAs of these data shall be discussed in detail here. In Figure 2a, the scores of *Corylus avellana* spectra feature negative values for the first and second PCs, which result in negative *Corylus-*typical peaks [15] in the respective loadings (e.g., at *m*/*z* 3098, *m*/*z* 4103 and the range from *m*/*z* 5400 to 6110 shown in Figure 3a, top black spectra and middle gray spectra). *Alnus cordata* spectra provide scores values that are positive for the first PC and negative for the second PC (Figure 2a). The corresponding loadings (Figure 3a) showed peaks in the lower mass range of *m*/*z* 1310 to 1463 and peaks around *m*/*z* 5204 and *m*/*z* 9488 that can be assigned to *Alnus* [15]. The *Pinus sylvestris* spectra were characterized by scores values close to zero for the first PC and very positive score values for the second PC. Consequently, the loadings of the second PC in Figure 3a showed peaks at *m*/*z* 4441 and *m*/*z* 4655 [15] that do not occur in the loadings of the first PC and that can be identified as *Pinus*-typical (compare with Figure A3a). The *Betula tatewakiana* scores values in Figure 2a were all close to zero, therefore the loadings showed no peaks that can be assigned to *Betula* pollen spectra.

In Figure 2h, *Corylus avellana* spectra yield negative scores values for the first PC and positive scores values for the second PC. Hence, the respective loadings (Figure 3h) showed negative and positive *Corylus*-typical peaks in the top black and the middle gray loadings, respectively (e.g., at *m*/*z* 4106 and the mass range from *m*/*z* 5400 to 6116). Furthermore, the *Alnus cordata* spectra in Figure 2h had zero values for the first PC and negative values for the second PC. Thus, in the loadings of the second PC in Figure 3h, negative values for the *Alnus*-typical peak pattern in the lower mass range (*m*/*z* 1313 to 1400 and peaks around *m*/*z* 1904) can be found (compare with Figure 1h). The *Pinus sylvestris* spectra featured positive scores values for the first and second PCs (Figure 2h), and *Pinus*-typical peaks at *m*/*z* 3944 and *m*/*z* 4658 can be observed in the corresponding loadings in Figure 3h, top black and middle gray loadings). *Betula tatewakiana* spectra in the PCA of Figure 2h, again, were characterized by scores values around zero, which result in loadings that did not show *Betula*-typical characteristics.

## 3. Discussion

In order to compare different preparation techniques for MALDI-TOF MS classification of pollen, we systematically varied the extraction procedure, the target material and the matrix solution. Our results demonstrated that higher absolute signal intensities could be recorded in the spectra obtained on conductive tape compared to those that were received with the same treatment strategies on the steel target (compare a–f of Figure 1 and Figure A1, Figure A2 and Figure A3 with g–l of the corresponding figure). Nevertheless, conducting PCA on data sets measured on a steel target was still expedient (Figure 2g–l). Kajiwara et al. [8] observed a slightly higher mass shift and a missing of some lower mass range peaks when mass spectra are obtained on conductive tape. These findings could not be confirmed by our experiments, where the usage of conductive tape as a sample holder facilitated the collection of mass spectra with intense peaks and less noise (a–f of Figure 1 and Figure A1, Figure A2 and Figure A3). We assume that the use of the sticky conductive tape could be favorable in the extraction by keeping the extracted analytes closer to the pollen grains, and therefore, enabling higher analyte concentrations that lead to more intense signals. The increased sensitivity could be especially important for the detection of single pollen grains in future applications. As shown in a previous investigation based on the MTP approach, MALDI MS is capable of detecting a few, down to ~3 pollen grains at best [7].

The treatment with acid seems to be indispensable for a sufficient extraction of analytes from the pollen grains (Figure 1 and Figure A1, Figure A2 and Figure A3). When no acid (formic acid or TFA, see d,j of Figure 1 and Figure A1, Figure A2 and Figure A3) is applied, the MALDI mass spectra exhibited less intense peaks and the different pollen species cannot be differentiated clearly in the PCA (Figure 2d,j). When a matrix solution without TFA was used, the PCA is dominated by the separation of *Corylus avellana* spectra from the spectra of the other species, most likely due to less noise and lower background in the *Corylus avellana* spectra. The other pollen species did not yield spectra of sufficient quality (see d–f,i–l of Figure 1, Figure A1 and Figure A3), leading to higher inner-class variances in the PCA. The addition of 1.25% TFA to the matrix solution led to an improvement in signal intensity and hence to higher quality spectra (a–c,g–i of Figure 1 and Figure A1, Figure A2 and Figure A3). As a consequence, the PCA of the data sets that were obtained with a matrix solution containing TFA most often enabled species-specific classification. The formic acid gas phase treatment resulted in intense signals from the birch pollen (Figure A1b), reasonable intensities for *Pinus* and *Alnus* pollen (Figure A3b and Figure 1b) and insufficient data for *Corylus* pollen (Figure A2b). Nevertheless, the PCAs of this dataset showed a clear intra-species homogeneity, even though the scores values of *Corylus* and *Betula*-spectra were almost alike. The extraction procedure conducted with formic acid droplet deposition obtained spectra with various intensities for each respective species resulting in large intra-group variance in the PCA. This might be traced back to the non-reproducible evaporation of the formic acid, caused by different droplet spot sizes.

## 4. Materials and Methods

### 4.1. Materials

In this study four different fresh pollen samples (*Pinus sylvestris*, *Betula tatewakiana*, *Alnus cordata*, *Corylus avellana*) of two plant orders (*Coniferales* and *Fagales*), collected from trees in the Botanic Garden of Berlin, were analyzed. After sample collection, the pollen grains were stored at −20 °C until usage.

### 4.2. Sample Preparation

For sample preparation, the pollen grains were deposited either separately on an MTP 384 standard target covered with double faced adhesive carbon tape (P77817, Science Services GmbH, Munich, Germany) or directly on a steel target. The following sample treatment procedures were compared using each target.

1. One microliter of HCCA matrix (10 mg of α-cyano-4-hydroxycinnamic acid diluted in 1 mL 1:1 acetonitrile/water (*v*/*v*) and 1.25% trifluoroacetic acid) was spotted onto the pollen grains.
2. Pollen grains were deposited on the target, which was kept for 3 min in a glass box over 98% formic acid (gas phase extraction). Afterwards, 1 µL of the matrix solution (see 1) was deposited.
3. One microliter of formic acid (98%) was pipetted onto the pollen grains. After drying at room temperature, 1 μL of the matrix solution (see 1) was added.

Additionally, these three procedures were repeated using a matrix solution without TFA.

After solvent evaporation, the target containing 48 spots was inserted into the mass spectrometer.

### 4.3. Data Acquisition

An Autoflex III MALDI-TOF mass spectrometer (Bruker Daltonik GmbH, Bremen, Germany) equipped with a 355 nm Smartbeam laser was used. Spectra were recorded in positive linear mode. Two thousand laser shots were accumulated for one spectrum. The laser settings were kept constant for all spots. From each spot five spectra were recorded at different positions. The instrument was calibrated using biopolymer standards (insulin, cytochrome C, myoglobin, and ubiquitin).

### 4.4. Data Analysis

The spectra pretreatment and multivariate analysis were performed using Matlab software (version R2015a, the Mathworks, Inc., Natick, MA, USA). Raw spectra were interpolated (reduction from 26,834 to 3634 data points) in the mass region between *m*/*z* 1100 and 12,000 with a step size of 3, baseline-corrected and vector-normalized. Subsequently, principal component analysis (PCA) of the samples that were obtained by the same pretreatment method was performed using the mass range between *m*/*z* 1100 and 12,000.

## 5. Conclusions

The following two preparation techniques were identified as especially suitable, since intense spectra were gained that featured characteristic mass peaks enabling a distinct classification in the PCA. In sample preparation (a in Figure 1 and Figure A1, Figure A2 and Figure A3) conductive tape was used as target, a mixture of ACN/H_2_O (1.25% TFA) served as matrix solvent, and no further extraction steps were conducted. The sample preparation (h in Figure 1 and Figure A1, Figure A2 and Figure A3) was pursued directly on the steel target, with gaseous formic acid extraction and TFA in the matrix solution. However, preparation (a in Figure 1 and Figure A1, Figure A2 and Figure A3) is by far the simplest sample preparation procedure; the pollen grains can be fixed easily on the conductive tape, and no further extraction steps must follow. Therefore, we suggest this procedure to establish a preoperatively simple and reliable routine method for pollen analysis based on MALDI-TOF MS. Future work will focus on the improvement of the sensitivity of this technique and the combination of MALDI imaging with multivariate data analysis.
